# Book Review

**Published:** 1921-06

**Authors:** 


### Reviews of Books.

**Functional Mental Illnesses, and the interdependence of the Sympathetic and Central Nervous Systems in relation to the Psycho-neuroses.** By R. G. ROWS, M.D., and DAVID ORR, M.D. Pp. 63. Edinburgh: Oliver and Boyd. 1920. Price 3s. 6d. These two papers, reprinted from the *Edinburgh Medical Journal*, will be read with interest by a wider circle of medical readers than might at first sight be suggested by the title under which they are published.

For whilst the centre of their subject is the inter-relationship between the emotions, the glands of internal secretion and the sympathetic system, their purport is a plea for the consideration of the sick man as a whole, and especially an insistence that "the whole nervous system, central and vegetative, constitutes one physiological unity," and that "the tone and excitability of the whole sympathetic system is regulated by the gland secretions or hormones which are poured into the blood. . . ."

The young general practitioner *au courant* with recent physiology equally with the matured of clinical experience and ripe for reflexion will find these papers stimulating. The general-physician, the physiologist, the pathologist, the psychotherapist and the alienist will read them with interest and find food for thought therein.

The important work of Professor Pavlov on the conditioned reflex is dealt with at some length (pp. 17–20), and further elucidated by clinical illustrations in the second lecture; but the very suggestive relationship between this knowledge and the treatment of " functional fits " does not receive the author's consideration.

The principle of " facilitation "—habit—is interestingly dealt with and illustrated with clinical examples.

The absence of definitions in the lectures of Dr. Rows must be conceded to be a defect, especially of " consciousness."

Concluding his lectures, Dr. Rows observes, " The type of result produced will depend not only on the suggestion adopted but also on the mental tendencies the patient has developed from the time of birth onwards, and on the sensibility and reactivity of his nervous mechanisms," and deplores the obscurity cast over the study of " functional mental illnesses " by concentrating upon the symptomatology of secondary phenomena.

It is not clear why dementia præcox should have been singled out from a long list of such phenomena for the attachment of the epithet " so-called."

Two interesting lectures, perhaps a little prematurely dogmatic in a few places, end with a plea for early treatment to prevent the appearance of the secondary phenomena which form the symptoms of a psychosis.

Dr. Orr's attitude is well illustrated by the concluding paragraph of his paper: " We must take a much broader view of the etiology and development of the psycho-neuroses than we have done hitherto. We can no longer regard them as psychic disturbances in the manner and sense employed in the past, and must be prepared in future to attach less importance to the ' psyche ' and approach the problem much more from the physiological side, as the inter-action of the ' psyche ' and the endocrino-sympathetic system under emotion is so very obvious " (p. 63).

There is a certain hardiness in " the determination of individual reactivity to an exciting cause, or in other words, temperament " (p. 58), but it is at any rate simple and straightforward.

Again, one feels that a definition of " consciousness " is needed that the reader may apprehend. " It must be granted that the will and consciousness have normally no influence on the vegetative system."

With these few points of criticism Dr. Orr's paper is clear and vigorous and maintains the reader's interest throughout.

The two papers are complementary, and this brochure (of 63 well-printed pages) forms a contribution to medical literature which should stimulate the thought and work both of clinicians and those engaged in research in a number of diverse fields of medicine.

**Malaria at Home and Abroad.** By Lieut.-Col. S. P. JAMES, M.D., D.P.H. (I.M.S., retired). Illustrated. Pp. xi., 234. London: John Bale, Sons & Danielsson Ltd. 1920. Price 25s. net.—The author's large experience as an administrator and teacher is a sufficient guarantee of the value of this attractive and well-illustrated book. Sir Patrick Manson when asked to write a "Foreword" said, "It requires no foreword from me or anyone else, it speaks for itself."

The book, while not pretending to be a complete treatise, is remarkably full of practical detail, and there is not a page in it which does not contribute in some way to the practical problems of the cure, control and eradication of the disease. This, we think, will be the reason of the strong appeal it will certainly make to medical men, whether their practice lies in the tropics or at home. Colonel James gives an interesting account of recent malarial outbreaks in England, and the measures to be adopted to cope with them. There is an excellent clinical section in which the varieties of the disease as observed at various ages and in various degrees of severity are described. Diagnosis and treatment are adequately dealt with, an important point, considering how much space is necessarily given to preventive methods. We note with much approval the emphasis which the author lays upon the necessity for microscopical work in connection with malaria, and the importance of thorough training and experience in this branch of investigation for all who intend to practise in the tropics. The book is well written, well illustrated and well got up, and deserves to become exceedingly popular with students and practitioners alike.

**A Text-Book of Pharmacology and Medical Treatment for Nurses.** By J. M. FORTESCUE-BRICKDALE, M.A., M.D. Pp. xv., 392. London: Oxford Medical Publications. 1920. Price 25s. net.—The author of any elementary text-book has many difficulties to contend with, and not the least of these is that of combining clearness of expression with accuracy of fundamental facts.

Many writers succeed quite well in making their subject easy to understand, but unfortunately the truth of their statements is often open to question. Dr. Brickdale, whose death has left the profession poorer in the domain of letters as well as of science, put his book forth in the plain, straightforward way that gave his writings such charm, while at the same time he spared no pains to ensure that facts were not misstated.

It is no easy task to write a book on pharmacology and treatment which is intended to be read by those who are well versed in the elements of chemistry and physiology, but in the present case the difficulty is enhanced, since nurses have seldom had the opportunity of acquiring much knowledge of these subjects. Nevertheless, the section dealing with chemistry is very clear, and should be readily understood by beginners; modern ideas are well brought forward, and the theory of ionization is dealt with in a simple and accurate manner. Even the very young science of colloidal chemistry receives some attention.

The section on pharmacology is illustrated by well-selected diagrams, and is thoroughly up to date, endocrinology and serology being dealt with in an admirable manner.

That part of the book devoted to treatment deals fully with general principles and also in greater detail with the methods adopted in treating a number of carefully-selected diseases.

Altogether we consider that the book well maintains the high standard of the series to which it belongs, and we commend it to those nurses who wish to excel in their profession.

**Anatomy: Descriptive and Applied.** By HENRY GRAY. Twenty-first Edition. Edited by ROBERT HOWDEN, M.A., M.B., D.Sc. Pp. xvi., 1,366. London: Longmans, Green and Co., 1920.—Although in the past decade the descriptive story of the pure anatomy of the dissecting room has changed but little, yet this edition is a great advance in the manner of its presentation and appeal to the student.

In the early pages of the book there is an up-to-date description of our advancing knowledge of human embryology, which offers a more and more enchanting and illuminating peep behind the ante-natal construction hoardings of man. In this chapter is revealed his primitive origin, and atavistic traits are explained.

The authors evidently realise that illustrations crystallise the information provided by the text, and diagrams are the keynote to the improvements of the last ten years.

In the chapter on joints the comparison of the relation between epiphyseal lines and the attachment of capsular ligaments is particularly commendable, also the extra-articular situation of the cruciate ligaments of the knee-joint. Very clear are the diagrams of the cardiac neuro-muscular mechanism.

The supremely complex connections of the ganglia of the autonomic nervous system with the cranial nerves are quite easily followed, thanks to numerous and lucid schemes of the nerve paths.

The book concludes with a collection of the surface markings of the whole body very clearly pictured, thus completing a work which is a triumph of precise science and pleasing draughtsmanship, and likely to preserve its premier position as a manual of Anatomy.

A lonely erratum appears on page 26, line 13, where apparently the printing of ulna instead of tibia has escaped notice.

**A Dictionary of Treatment, including Medical and Surgical Therapeutics.** By Sir William Whitla, M.A., M.D., LL.D., M.P. Sixth Edition. Pp. viii., 1,083. London: Baillière, Tindall and Cox. 1920. Price 25s. net.—A revised edition of this favourite work will be heartily welcomed, and the strain of Parliamentary duties has not prevented the indefatigable author from carrying out active revision.

The surgical articles have been entrusted to Mr. S. T. Irwin, the ophthalmological to Wiclif McCready, and the gynæcological to R. J. Johnstone, and an enormous amount of information has been collected in the thousand large pages of this edition. A curious misprint occurs in the article on pneumonia, where 12 c.c. of a 1 in 5 solution of camphor are spoken of as a hypodermic dose. We could wish that a full account of the fasting treatment of diabetes were given and the Epstein diet in tubal nephritis; but one is astonished at the number of useful methods which are included. Sir William's criticism and discussion of various modes of treatment is not the least valuable part of the book.

**The Diagnosis of Nervous Diseases.** By Sir James Purves Stewart, K.C.M.G., C.B. Pp. viii., 584. Illustrated. London: Edward Arnold. 1920. Fifth Edition. Price 30s. net.—It is unnecessary to lay stress on the usefulness and value of this well-known work, or to point out the features which are so well known to a large circle of readers.

The fifth edition has been revised and in part re-written, so that while those who have not yet availed themselves of the knowledge and experience stored in previous editions may be confidently recommended to begin on this one, those who have valued the book in the past will find that the present volume supplies them with that residuum of recent information which they need, and should certainly not miss the opportunity of acquiring. Quite apart from the value of the text, the excellent way in which the book is produced and the numerous coloured plates and illustrations make it a remarkably cheap volume at the price.

**Infectious Diseases.** By Claude Buchanan Ker, M.D., F.R.C.P. Ed. Second Edition. Pp. xii., 625. Oxford Medical Publications. 1920.—This is an excellent book, which deals with the problem of the infectious diseases in a thoroughly practical manner. The main outstanding features of the work are the methods of diagnosis, prognosis and treatment, each of these being considered in complete detail. The wide personal experience of the author is of the greatest value. The practitioner will therefore find this book an invaluable help with regard to the detailed management of cases. Pathology occupies a relatively subsidiary position. Owing to the large amount of fresh knowledge which has been acquired since the appearance of the first edition, several portions of the book have been entirely re-written and there are numerous alterations and additions. The illustrations, many of which are coloured, demonstrate remarkably clearly the details desired. There are also very numerous charts. The last chapter is devoted to the problems of Fever Hospital Management.

**Essentials of Tropical Medicine.** By Walter E. Masters, M.D. Pp. vii., 702. London: John Bale, Sons and Danielsson, Ltd. 1920. Price 42s. net.—To those preparing for examinations and to the busy tropical practitioner the author has rendered a distinct service in compiling a synopsis of the salient facts of Tropical Medicine. The first four sections devoted to Tropical Medicines proper are classified according to etiology; the remainder to such diverse subjects as Venoms and Poisons, diseases of the Skin and Eyes, Tropical Hygiene. That such a book should comprise 700 pages is of itself witness to the enormous increase in our knowledge of this special branch of medicine. The use of large type, generously spaced, makes the volume bulky. An excellent feature is the summary of the historical facts in dealing with the more important diseases. The details of treatment and prophylaxis are given very fully. It is manifestly difficult to epitomise the salient features of differential diagnosis in a line. This is often attempted by the author under the heading of diagnosis, and in our opinion it leads him in several instances to make positive misstatements. Later, on page 606, we read that "in lymphatic leukæmia . . . the leucocytes are increased to 14,000 per cm."; "in spleno-medullary leukæmia the red cells are reduced to 700,000 per cm."!

**Functional Nerve Disease.** Edited by H. Crichton Miller, M.A., M.D. Pp. xi., 208. London: Oxford Medical Publications. 1920. Price 8s. 6d. net.—This little volume of essays by different writers is an attempt to set forth the new psychogenetic concept of functional nervous disorders as it has been revealed to us during the war, so that the users may find application to problems of peace time. To write a text book of the subject is as yet beyond the wit of man; first, because the field to be covered is so vast and so little explored; and second, because it is almost impossible to write of these psychological processes without falling into a slang which is as distressing to the eye as it is perplexing to the intellect. The writers of these essays have not quite avoided the use of this jargon, but they are obviously doing their best to be intelligible, and they have succeeded far better than most of those who write on such subjects. The lessons which the book teaches enter into every branch of medicine, and are destined to effect a revolution in practice at least as profound as the discovery of anæsthesia. We wish the book the wide circulation which it deserves.

**An Atlas of the Primary and Cutaneous Lesions of Acquired Syphilis in the Male.** By CHARLES F. WHITE, M.B. and W. HERBERT BROWN, M.D. Pp. vii., 32. 35 Plates. London: John Bale, Sons and Danielsson, Ltd. 1920. Price 27s. 6d. net.—This Atlas comes at a time when venereal diseases are occupying a large amount of attention in the medical as well as the civilian world. With many excellent photographs (some stereoscopic), and a number of beautifully-coloured drawings of the various venereal lesions, it gives a good representation of the different forms these infections may take, and should certainly assist materially students and practitioners in diagnosis. The plates are prepared by a skilled artist from cases carefully selected from a very large quantity of clinical material, and should carry out the chief aim of the authors, viz. "to give assistance in early diagnosis." The chapter on Primary Syphilitic Sores is excellent, and this alone would recommend the Atlas to anyone specially interested in venereal disease, as well as to the student. The last chapter, on "Diseases not infrequently mistaken for Syphilis," might well be amplified, and it is to be hoped that future editions may be forthcoming in an enlarged form.

**Air Sickness.** By CRUCHET and MOULINIER. Translated by J. ROSSLYN EARP, M.A., M.R.C.S., Pp. xv., 96. London: John Bale, Sons and Danielsson, Ltd. 1920. Price 5s. net.—This interesting little essay is founded on certain observations made by the authors before the war and since somewhat simplified. They are disciples of Professor Pachon of Bordeaux, and have applied his celebrated oscillometer to the elucidation of the symptoms observed in airmen during and after their flight. Many charts showing maximal and minimal arterial pressure are given, and the authors attribute most of the unpleasant, or even dangerous, symptoms to vasomotor changes. An interesting series of similar observations on athletics in other fields is given and the results compared with those found in flying men. The book will be read with advantage, therefore, by all who are interested in the physiological results of strenuous exercise and in tests for physical fitness generally. Dr. Pachon contributes a preface, and wing-commander Martin Flack an introduction to the English translation. It should be added that the translator has done his work adequately and pleasantly, though an occasional Gallicism crops up here and there.

**The Doctor's Manual.** By A. Herbert Hart, M.S. Pp. xv., 256. London: John Bale, Sons and Danielsson, Ltd. 1920. Fourth edition. Price 10s. 6d. net.—The fact that this is a fourth edition seems to indicate that the book finds interested readers. Probably the first sixty pages, containing a list of stock medicines for the dispensary, with certain useful hints and directions based on practical considerations, are the most appealing part of the book. Each mixture has a sort of pet name, denoted by capital letters, which are not initials in all cases (thus *Liquor Atropinæ Sulphatis* is called L.A.P.), and which seem to be as difficult to remember as the full names. A little more care might have been taken to avoid errors in the Latin words used, which are, moreover, freely mingled with English in the formulæ. Thus "Injectio Hypodermicæ" occurs in the Table of Contents, "Pigmentæ" on page 55, "Unguentum Tinea" on page 60, among other examples. The next 120 pages are devoted to proprietary drugs and foods and descriptions of spas and mineral waters. The style is reminiscent of much of the "Literature" on these subjects, which can usually be obtained free of cost. The remainder of the book contains a "Synopsis of Treatment," which does not differ materially from other similar attempts to condense a complicated subject into a few short paragraphs; and finally an article on Venereal Disease by Mr. Frank Kidd, whose portrait adorns the title-page.

**A Medical Handbook, for the use of Practitioners and Students.** By R. S. Aitchison, M.D. Fifth edition. Pp. xvi., 390. London: Charles Griffin and Company, Ltd. 1920. Price 10s. 6d. net.—The first edition of this book appeared in 1893. The present is its fifth edition, which, according to the preface, has been carefully revised and brought up to date. That a hand-book embodying a close and balanced summary of the whole field of modern medicine can be written is demonstrated by this work; but the past twenty years have wrought such change in our view of disease that it seems almost impossible to bring a book first written in 1893 up to date without recasting its entire contents. Yet the author has succeeded in compressing an enormous mass of information into a small compass and into a readable form, and it would hardly be fair to cavil at omissions.

**The Bradshaw Lecture on the Surgery of the Heart.** By Sir Charles A. Ballance, K.C.M.G., C.B., M.V.O., M.S. London. Pp. 154. London: Macmillan and Co., Ltd. 1920. Price 10s. 6d. net.—Apart from war wounds the knowledge of most surgeons of this subject is limited to the treatment of pericardial exudations, for as its author tells us, in the words of Guthrie, sensible people are not "disposed to stab each other while madmen try to kill themselves in a different way."

After referring to various ancient and modern views of the surgery of the heart, we are told that Farina sutured a wound in the left ventricle in 1886, and that the "first suture of a wound in the auricle was done in 1898 by Giordano." The author considers that the operation of paracentesis pericardii should be "banished from surgical practice," and would substitute exposure of the pericardium by removal of the sternal ends of the left sixth and seventh cartilages. In this he appears to have gone ahead of general opinion, and it is scarcely a fair comparison to instance the undesirability of puncturing the intestines in a case of obstruction in support of his contention. Paracentesis of the right auricle and ventricle is another matter, and those of us who have seen this done will agree with the remarks as to its dangerous character, and be content with free bleeding from the external jugular in such cases instead.

Quoting various statistics of heart wounds, it is shown that more than one-third get well after operation, whereas in the pre-operation period only 15 per cent. of cases recovered. The various operations are well described, and the book contains forty-eight illustrations. The opinions and work of other surgeons has been ably collected, and the lecture clearly presents to us practically all that is known of the operative possibilities of a region which has only comparatively recently been regarded as within the limits of legitimate surgery.

**Operative Surgery.** By Alexis Thomson, F.R.C.S. Edin., and Alexander Miles, F.R.C.S. Edin. Third edition. Pp. xviii., 619. London: Henry Frowde, Hodder and Stoughton. 1920. Price 16s. net.—This *Operative Surgery* is a companion volume to the author's *Manual of Surgery*. In the present edition the subject-matter has been largely re-written and expanded. The descriptions of operations are concise, and the illustrations appended are numerous and helpful. Minute technical details are beyond the scope of the work. We commend it as an excellent introduction to the practice of Operative Surgery and as an excellent text-book to use in preparing for ordinary surgical examinations.

